# Minnesota State Veterinary Medical Association

**Published:** 1900-09

**Authors:** K. J. McKenzie

**Affiliations:** Secretary-Treasurer


### SOCIETY PROCEEDINGS.

### MINNESOTA STATE VETERINARY MEDICAL ASSOCIATION.

MINNEAPOLIS, MINN., JULY 11, 1900.

The seventh semi-annual meeting was called to order by the President, S. H. Ward, at 10 A. M., at the Holmes Hotel, Minneapolis. The following members responded to roll-call: Drs. C. C. Lyford, S. D. Brimhall, J. G. Annand, A. A. Keyes, J. S. Butler, and M. J. Saxton, of Minneapolis; M. H. Reynolds, St. Anthony Park; L. Hay, Faribault; K. J. McKenzie, Northfield; S. H. Ward, St. Cloud; W. Amos, Owatonna; J. N. Gould, Worthington; A. F. Lees, Red Wing; H. C. Lyons, Hutchinson; H. C. Peters, Litchfield; W. A. Sproule, Waseca; T. Falconer, Glenwood.

The minutes of the previous meeting were read and accepted. The Treasurer's report was read and accepted. The following applicants were balloted upon and accepted as members of the Association: Drs. J. G. Harris, J. W. Cook, and J. McKay, of Duluth; Drs. R. Lapointe and J. Murray, of Le Sueur, Dr. D. M. McDonald, of Brainerd; Dr. J. P. Foster, of Bangor, S. D.

Communications from Drs. F. H. Farmer, M. S. Whitcomb, and B. Lambrechts, were then read, each regretting his inability to attend.

The meeting then adjourned for dinner.

AFTERNOON SESSION, 1.30 O'CLOCK.

Dr. W. Amos gave his report on veterinary colleges, bringing out some important facts in connection with some of our veterinary colleges, also stated that two new veterinary colleges had been instituted since our last meeting.

Dr. C. C. Lyford reported for the Committee on Legislation and Empirics. The doctor read a letter from the County Attorney of Hennepin County, stating that in his opinion the law regulating the practice of veterinary medicine and surgery in this State was valid and all right, but still did not advise carrying the McCulloch case to the Supreme Court. At this juncture a Mr. Mercer, one of Minneapolis' most prominent attorneys, was called in to explain a few points in regard to the wording of the law. A lengthy discussion followed. On motion it was decided to drop the case against Dr. McCulloch and not carry it to the Supreme Court, but instead, that a new case be brought to fit under Section 8 of the law, in Ramsey County, and that a new committee be appointed to look after same. Committee appointed was: Drs. R. Price, B. A. Pomeroy, G. A. Dallamore.

Dr. S. D. Brimhall next reported for Committee on Infectious Diseases, stating the number of cases of glanders reported since last meeting, number quarantined, number tested, and number condemned. Also number of cases of tuberculosis reported, number tested, and number condemned. The doctor reported two outbreaks of anthrax. Malignant catarrh had been reported from three districts, being from Steele, Beecker, and Blue Earth counties. Blackleg seemed scattered through a good many different parts of the State.

Dr. Sexton reported for the Committee on Finances. A communication was read from Dr. R. Price, who was then at St. Luke's Hospital, in St. Paul, in which he gave his experience on the operating-table while being operated upon for varicose veins. Dr. Reynolds gave a lengthy report on recent advances in veterinary surgery, which was both interesting and instructive. Dr. S. D. Brimhall gave an extended report on recent veterinary literature, touching on bacteriology. Dr. J. N. Gould reported recent veterinary literature bearing upon medicine.

The meeting then adjourned for supper, after which several autopsies were held on glandered horses, at Carlson Bros., on First Avenue, North. The balance of the evening was spent in the discussion of glanders and tuberculosis and in the report of cases.

THURSDAY, JULY 12TH, 9 A.M.

The members met at Dr. C. C. Lyford's Infirmary for the following clinics: Plantar neurectomy, Dr. L. Hay and Dr. J. G. Annand; castration of a cryptorchid, Dr. J. N. Gould; plantar tenotomy, Dr. K. J. McKenzie; operation for capped hock, Dr. C. C. Lyford.

Thursday afternoon was spent at Lake Minnetonka, where the Twin City members had generously provided an outing for the rest of the members and their wives. The first-class steamboat, "The Victor," having been previously chartered for the afternoon, we were given a fine boat-ride; supper was served on the boat. This was considered a good way to bring the members of the veterinary profession and their families together so that they might become better acquainted. To say that a pleasant afternoon was spent does not express it; each returned to his home feeling that his time had been pleasantly and profitably spent at this meeting.

K. J. MCKENZIE,
Secretary-Treasurer.

